# Supplementary material for: Effects of Mixed Pasture Legume Phytoestrogens on Superovulatory Response and Embryo Quality in Angus Cows
Source: Animals (Basel). 2024 Apr 7;14(7):1125. doi: 10.3390/ani14071125 (PMC11011016; doi:10.3390/ani14071125)
Supplement: Supplementary file 1 [file animals-14-01125-s001.zip › Supplementary Table S1.pdf]

Supplementary Materials: The following supporting information can be downloaded at: www.mdpi.com/xxx/s1, Figure S1: title; Table S1: title; Video S1: title.

Table S1. Dynamic multiple reaction mode (dMRM) parameters used for quantifying coumestrol and selected isoflavones in plasma and pasture samples via UHPLC-MS-QQQ. All compounds used an acceleration voltage of 5 eV.

Compound	Precursor ion *(m/z)*	Product ion *(m/z)*	Retention time (min)	Retention time window (min)	Fragmentor voltage (eV)	Collision energy (eV)
daidzein	253	132	5.9	0.8	135	55
genistein	269	133	8.1	0.7	135	40
coumestrol	267	91	8.37	0.5	135	50
formononetin	267	251	9.3	0.6	135	35
biochanin A	283	268	11.1	0.6	135	30
